# Storage Stability and Sensory Properties of Raha Sweet Colored with Crude and Purified Red Grape Anthocyanins and Synthetic Food Colorant

**DOI:** 10.3390/foods13172747

**Published:** 2024-08-29

**Authors:** Ayed Amr, Sarah Jaradat, Ashraf Al-Khamaiseh, Salameh Alqaraleh, Haneen Tarawneh, Samah AlBataineh, Imad Hamadneh, Hatim AlKhatib, Mohammad Shahein

**Affiliations:** 1 Department of Nutrition and Food Science, The University of Jordan, Amman 11941, Jordan; ayedamr@ju.edu.jo (A.A.); salamehalqaraleh@yahoo.com (S.A.); 2 Department of Clinical Nutrition and Dietetics, Applied Science Private University, Amman 11941, Jordan; a_khamaiseh@asu.edu.jo; 3 Department of Food and Nutrition, Jerash University, Jerash 26110, Jordan; h.tarawneh@jpu.edu.jo (H.T.); s.batineh@jpu.edu.jo (S.A.); 4 Department of Chemistry, The University of Jordan, Amman 11941, Jordan; i.hamadneh@ju.edu.jo; 5 Department of Pharmaceutics and Pharmaceutical Technology, The University of Jordan, Amman 11941, Jordan; h.khatib@ju.edu.jo; 6 Department of Medical Allied Sciences, Zarqa College, Al-Balqa Applied University, Zarqa 13110, Jordan; mohammad.shaheen@bau.edu.jo

**Keywords:** anthocyanins, grapes, Raha Sweet, pigments, half-life, storage stability, kinetics

## Abstract

Anthocyanins (ANCs) are water-soluble pigments that are useful as nutraceuticals due to their health benefits. This study was performed to evaluate the storage stability of purified and crude red grape ANCs in Raha Sweet (RS) during storage and to evaluate its sensory properties. ANCs were extracted from red grape pomace and purified with a macroporous resin. RS was prepared and colored with a synthetic food dye, Carmoisine (control), and ANCs (crude and purified). Pigments were extracted from RS weekly for a period of seven weeks and the absorbance was read spectrophotometrically. RS colored with ANCs was evaluated for its color and other sensory properties against another RS colored with the control. Results showed that the degradation of ANCs in RS followed the first-order reaction model, unlike the control, which showed no degradation during storage. The half-life of crude ANCs was three times higher than that of the purified ones, and RS colored with ANCs received a significantly (*p* < 0.05) lower score for color than that of RS colored with the control. ANCs could provide the food industry with a natural alternative to synthetic dyes to color foods with high sugar content that are stored for a short period of time.

## 1. Introduction

Natural and synthetic colorants are used as food additives to enhance the appearance of food products and attract consumers. Although synthetic food colorants have lower production costs and higher stability and usability, some studies have emphasized the strong relationship between them and their adverse health effects [1]. As a result, the demand for natural food colorants has increased rapidly in the international market since they are non-toxic and environmentally friendly. Natural food pigments are easily extracted and generally obtained from plants, insects, animals, and minerals [2].

The most recognizable natural colorants to date are betalains, anthocyanins (ANCs), caramels, carmine, carotenoids, curcumin, and riboflavin [3]. ANCs represent a potential alternative to synthetic dyes due to their appealing color and possible health benefits. The use of ANCs as a food coloring has been approved and encouraged [4] in several countries (E163). In addition to imparting color to foods, they may act as antioxidants and as free radical scavengers to protect the food to which they are added, and they possess anti-inflammatory and anticarcinogenic activities [5], therefore providing added value to food. ANCs appear in different colors depending on the pH of the surrounding environment; in acidic media (pH 1–3.5), they assume a red color which fades gradually until reaching pH 4, where they become colorless. At near-neutral pH, ANCs start to appear bluish-purple, whereas at pH above 8, a yellow color appears [6].

Red grapes, *Vitis vinifera*, are rich sources of ANCs which give the unique color to red wine and grape juice. However, a considerable amount of ANCs are lost during wine making and juice production in the form of skin and pomace [7]. Yet, grape ANCs are some of the least stable because they lack acylation [8]. Given that acylated ANCs are more stable than their non-acylated counterparts, acylated ANCs are often used as food colorants in the food industry [9]. Acylated ANCs are present in flowers and vegetables such as purple sweet potato, red radish, purple and black carrot, red cabbage, and purple corn, whereas non-acylated ones are found in fruits such as grapes, blackberries, blackcurrants, blueberries, and elderberries [10].

Raha Sweet (RS), also known as Turkish delight, is a gel-like dessert made of sugar, starch, and water as the main ingredients, flavored with rose water or other scents and colored with fruit concentrates or synthetic dyes. Citric acid or lemon juice are often added to prevent the crystallization of sugar [11]. RS is one of the world’s oldest desserts, with a history spanning over 600 years. RS is known under different names in Eastern Europe and the Middle East: lokum in Turkey, Rahat-alhulkum in the Arab World, lokun in Bulgaria and Macedonia, loukoumi in Cyprus and Greece, ratluk in Bosnia and Herzegovina, and rahat in Romania. It was first introduced to Western Europe in the 19th century by an Englishman who became dazzled with lokum during his visit to Turkey, where he bought some and sent it across to England. Since then, RS has become famous across the Western world [12].

RS has been previously colored with black carrot juice concentrate [12], and red grape and sour cherry concentrates [13]. However, RS in those studies was not colored with extracted ANCs added as such to the other ingredients. In addition, data on the investigation of the spectral properties of purified grape ANCs in relation to different pH conditions and their use in the coloration of food products, especially RS, are not available. Therefore, this study aimed to examine the effect of pH variation on the spectral properties of ANCs extracted from red grape pomace, investigate the storage stability of purified and crude red grape pomace ANCs in RS during seven weeks of storage, and evaluate the sensory properties of RS in terms of color, odor, taste, texture, and overall acceptability.

## 2. Materials and Methods

### 2.1. Plant Material and Chemicals

Fresh red grapes (Black Magic) were purchased from a local grocery in Amman, Jordan. Grape berries were detached from stems and washed with distilled water. Grape berries were removed manually from seeds and the resulting pomace was stored in a freezer in plastic bags until extraction. All chemical reagents were purchased from a local distributor in Amman, Jordan.

### 2.2. ANCs Extraction

ANCs were extracted from red grape pomace according to [14]. Briefly, red grape pomace was thawed at ambient temperature, put onto a tray, and dried in an oven (Memmert GmbH, Schwabach, Germany) at 45 °C. Dried pomace was ground with a coffee grinder (Bosch, Gerlingen, Germany) and sieved through a 600-micron mesh sieve. The obtained fine particles were mixed with acidified aqueous ethanol (50% *v*/*v*, 1% citric acid) using a magnetic stirrer for half an hour and vacuum filtered. The process was repeated until the color of the filtrate became colorless. Afterwards, the fractions were combined, and the solvent was evaporated under reduced pressure using a rotary evaporator (Buchi, Flawil, Switzerland). The resulting concentrate was reconstituted with distilled water, centrifuged at 8000 rpm for 10 min (Hermle, Gosheim, Germany), placed in a screw-capped flask after flushing with nitrogen, and stored in a freezer until purification.

### 2.3. ANCs Purification

The macroporous resin, Diaion HP-20 (Sigma-Aldrich Co., St. Louis, MO, USA), was soaked in aqueous methanol (50% *v*:*v*) for three days, after which it was loaded into a 30 cm glass column. The crude ANCs extract was loaded into a glass column partially filled with the resin and washed several times with distilled water to remove sugars and other highly water-soluble compounds, until the eluted water became colorless. Afterwards, acidified aqueous ethanol (50%) was used to eluate the ANCs. The solvent was evaporated with a rotary evaporator and the resulting concentrate was diluted with distilled water and centrifuged at 8000 rpm for 5 min. The supernatant was taken and stored in a freezer until further usage.

### 2.4. Quantification of Monomeric ANCs

Monomeric ANCs were quantified using the pH differential method of [15] as in Equation (1)

(1) $$\mathrm{Total}\;\mathrm{ANCs}\;\mathrm{content}=(\frac{\mathrm{mg}}{L})=\frac{A\times \mathrm{MW}\times \mathrm{DF}\times 10^{3}}{\varepsilon }\;A=\left(A_{520\mathrm{nm}}-A_{700\mathrm{nm}}\right)\;\mathrm{pH}\;1.0-\left(A_{520\mathrm{nm}}-A_{700\mathrm{nm}}\right)\;\mathrm{pH}\;4.5$$

where *A* is the absorbance of ANCs, *A*_520nm_ is *A* at 520 nm, and *A*_700nm_ is *A* at 700 nm. MW is the molecular weight of cyanidin-3-glucoside (449.2 g/mol), DF is the dilution factor, 10^3^ is the factor used to convert g to mg, and ε is the molar extinction coefficient of cyanidin-3-glucoside (26,900 L/mol·cm).

Monomeric ANCs were expressed as cyanidin-3-glucoside equivalents in mg/L.

### 2.5. Spectral Changes of ANCs at Different pH Values

To understand the effect of pH variation on the color and spectral properties of purified ANCs, one part purified ANCs was diluted with four parts of citrate-phosphate buffer solution (0.1 M citric acid monohydrate + 0.2 M disodium hydrogen phosphate) at different pH values (2, 3, 4, 5, 6, 7, 8, and 9) and incubated at room temperature for 30 min before absorbance was taken using deionized water as a blank. Spectral properties in terms of color, λ_max,_ and the corresponding absorbance were recorded at each pH value using an ultraviolet–visible spectrophotometer (Varian Carry, NSW, Australia).

### 2.6. The Use of ANCs in the Coloration of RS

#### 2.6.1. Preparation of RS

**Figure 1:**
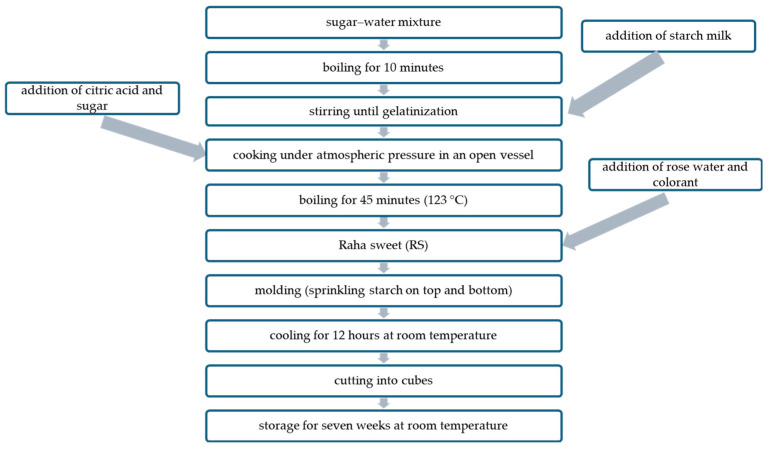
Flow diagram of RS preparation.

RS was prepared as follows (Figure 1). Water (750 g) was mixed with table sugar (400 g) (sugar syrup), placed in a cooking vessel, and stirred with heating until boiling. Starch (200 g) was mixed with water (250 g) (starch milk), added to the water–sugar mixture, and stirred with heating until gelatinization. Citric acid (10 g) was added, followed by another sugar portion (400 g). The resulting mixture was left to boil for 45 min with occasional stirring in an open vessel, to which rose water (50 g) and pigments (100 g) (purified and unpurified ANCs and the synthetic red food dye (control)) were added separately at 123 °C. The mixture was poured into a plate and left overnight after sprinkling starch on the top. Afterward, RS was cut into cubes, sprinkled with starch, and kept in an airtight container for 49 days. The percentage of different ingredients in RS is presented in Table 1.

#### 2.6.2. Storage Stability of ANCs in RS

Briefly, 30 g of RS colored with ANCs, or the control, was mixed with 150 mL of acidified methanol (0.1% HCl) and homogenized in a blender (Moulineaux, Normandy, France) until obtaining a homogeneous mixture. The resultant homogenate was allowed to stand for 90 min in the dark at room temperature, after which the upper layer (colored) was separated from the lower one and centrifuged at 10,000 rpm for 10 min. The supernatant was taken and the corresponding absorbance was read using a spectrophotometer with methanol as a blank. The absorbance of ANCs and the control were taken on zero time, and this was repeated at one-week intervals until the end of the storage period (seven weeks). The results were expressed as pigment retention (PRT) in days as in Equation (2):

(2) $$PRT=\frac{Absorbance\;of\;pigment\;at\;any\;given\;time\;}{Absorbance\;of\;pigment\;at\;zero\;time}$$

#### 2.6.3. Sensory Evaluation of RS

Thirty-one individuals from both genders aged between 20 and 75 (all habitual consumers of this sweet) evaluated two RS samples (colored with ANCs versus the control) on a scale of 1–5 adapted from [13] for color, taste, texture, odor acceptability, and overall acceptability, where 1 = dislike very much, 2 = dislike, 3 = neither like nor dislike, 4 = like, and 5 = like very much.

Both samples were coded with random three-digit numbers and served with biscuits in squared plastic plates. Each panelist was asked to rinse his/her mouth with water between each sample tasting.

### 2.7. Statistical Analysis

Data were analyzed using the Statistical Package for Social Sciences (SPSS version 22.0, IBM Corp., Armonk, NY, USA). Differences among means were determined by the paired T-test and considered significant at *p* < 0.05 and values were presented as mean ± SD.

## 3. Results and Discussion

### 3.1. Effect of pH Variation on the Spectral Properties and Color of Purified ANCs

**Figure 2:**
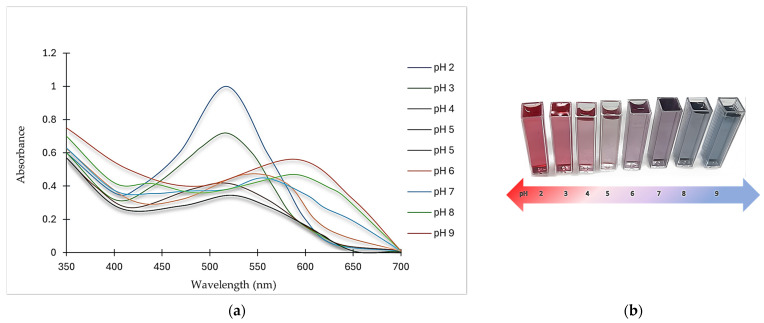
Effect of pH variation on (**a**) spectral properties and (**b**) color change of ANCs.

According to Figure 2 and Table 2, pH variation affected λ_max_, the relative absorbance, and the color of ANCs. In general, the relative absorbance and color intensity decreased as the pH increased up to a certain point. The absorbance was maximum at pH 2 (517 nm), assuming a pinkish-red color, and became less than three-quarters at pH 3 and less than the half at pH 4 relative to the maximum absorbance.

As the pH increased to 5, the absorbance fell to the minimum, reaching one-third of the maximum absorbance. Conversely, at pH 6, there was a slight bathochromic shift in λ_max_ where ANCs turned pinkish-violet (supported by the color profile in Figure 2b), and the absorbance rose again, amounting to almost half of that at pH 2. Another bathochromic shift emerged at pH 7, and the color shifted to a violet hue but with a slight decrease in λ_max_. Two consecutive bathochromic shifts in λ_max_ appeared at pH 8 and 9, where the absorbance at pH 8 increased slightly, reaching nearly the same relative absorbance of that at pH 6 with a change in the color from violet to bluish-purple. At pH 9, the bathochromic shift in λ_max_ generated a more distinct blue hue with an absorbance amounting to more than half of that at pH 2.

Generally, color intensity in highly acidic media was higher than in low acidic, neutral, and alkaline media. ANCs go through reversible structural changes when the pH changes, which changes their color [16].

For instance, butterfly pea flowers’ ANCs gradually changed color from magenta to purple, blue, and green as the pH increased from 0.5 to 9. ANCs appeared red (λ_max_ at 548 nm) at pH 0.5–3, and the intensity decreased as the pH increased. The color shifted from magenta to purple as the pH increased, and the absorption peak was divided into two peaks at 570 nm and 622 nm, with a shoulder peak at 531 nm. The color turned vivid blue at pH 5 to 8, and the peaks at 576 nm and 622 nm increased slightly as the pH increased, whereas at pH 9, another bathochromic shift emerged [17].

In another example, the UV–visible spectra of roselle ANCs in different pH buffers were studied and the maximum absorption peak was obtained at 520 nm in acidic media, where the absorbance gradually decreased as the pH increased, accompanied by a red shift in the visible spectrum [18]. While ANCs isolated from different sources have some color and intensity similarities in response to pH fluctuations, the color generated is mainly determined by the prevalent anthocyanidins in the plant matrix.

### 3.2. Degradation Kinetics of ANCs in RS during Storage

**Figure 3:**
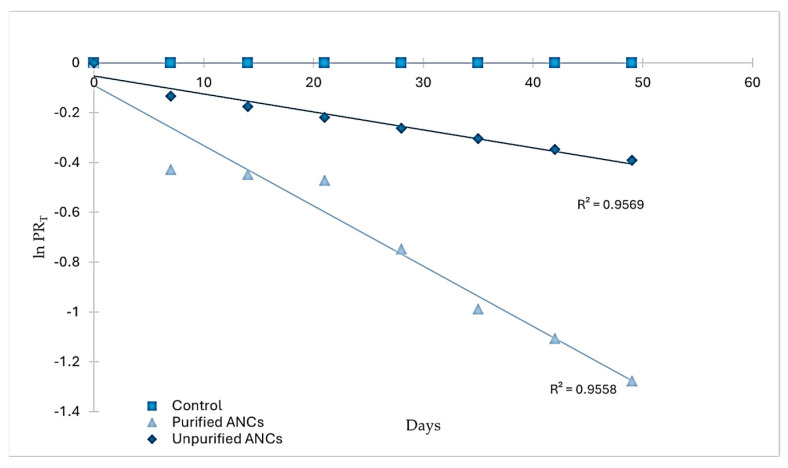
Degradation kinetics of ANCs and control in RS during storage.

RS was stored for seven weeks in airtight containers to study the degradation of pigments (ANCs and the control) during storage. The degradation of ANCs in RS followed the first-order reaction model when the natural logarithm of PRT was plotted vs. time (days) (Figure 3), which agrees with the storage stability of ANCs of black carrot juice concentrate in RS [12] and jam [19], and blackberry, raspberry, and cherry in jam [20].

The reaction rate constants (K) and the half-lives (t_1/2_) (Table 3) were calculated as in Equations (3) and (4)

(3) $$In\;PRT_{t}=K\;t+Ln\;PRT_{0}$$

where PRT_0_ is the initial PRT and PRT_t_ is the PRT after a given t (time in day).

(4) $$t\frac{1}{2}=\frac{0.693}{K}$$

An inverse relationship exists between the reaction rate constant and the half-life; that is, the higher the reaction rate constant, the lower the half-life and vice versa [21]. In RS, the estimated half-life of crude ANCs was 96.25 days, which was at least three times higher than that of the purified ones (28.64 days). The difference in the half-life between the two forms of ANCs in RS was due to the difference in the reaction rate constant between them. Meanwhile, the control exhibited no degradation during the storage period. In a related study, ANCs were much less stable than synthetic colorants (Carmoisine, Allura Red, and Ponceau) at higher temperatures, even though they were not added to food products [22]. Crude and purified ANC extracts may differ in their stability depending on the presence of other compounds in the crude extracts (sugars, other phenolic compounds, organic acids, salts, and others) that improve ANCs’ stability. According to two studies on some ANCs extracts, purified ANCs degraded faster than crude ANCs due to inter- and intramolecular co-pigmentation processes [23,24]. Therefore, RS with unpurified ANCs might have benefited from the presence of co-pigments in the extract, which may exert a protective effect on the storage stability of ANCs.

It is worth mentioning that grape ANCs were added at the end of RS manufacturing when the mixture reached an extremely high temperature, which might have accelerated ANCs degradation during storage. However, ANCs were heated for up to a minute during the last step of RS production. The short heating time to which ANCs were subjected may have limited the degradation process to the minimum.

For example, the impact of temperature on the degradation of ANCs from acerola pulp in actual heating conditions became considerable only for the long-time duration; ANCs loss in a model industrial pasteurization system was 1% at varied temperatures (60, 70, 80, and 90 °C) for 20 s [25]. A few authors reported that the duration of heating had a greater impact on ANC loss than temperature, as demonstrated by certain foods like bread [26].

It appears that the source of ANCs may influence their storage stability. The t ½ of ANCs varied noticeably between different sources when their storage stability in jam was studied [20]; wild cherry ANCs had the highest half-life, amounting to 157.2 days, followed by sweet cherry (108.1 days), blackberry (96.2 days), and red raspberry (74.2 days). It was shown that RS colored with black carrot juice concentrate was more stable at 20 °C (160.5 days) but less stable at 30 °C (80.25 days) [12] than RS with unpurified ANCs in the current study, which might be due to the acylation effect. Acylated ANCs are more stable than non-acylated ones [10].

On the other hand, cultivar could have an additional effect on the storage stability of ANCs. For example, it was reported that the half-life of ANCs during storage varied depending on the source cultivar when black carrot concentrate was utilized to color jam; the half-life of ANCs was 19.6 weeks in Osmanli cultivar and 21.5 weeks in Kara cultivar [19].

Separating heating effects from the food matrix’s effects is difficult [27]. In this work, adding sugar might have an additional protective effect on the thermal stability of ANCs. For example, sugar was shown to exert a protective effect on strawberry ANCs by reducing water activity and oxygen absorption [28], although other authors reported that sugar accelerated the degradation of Concord grape and amaranth ANCs [29,30] and Ranunculus asiaticus ANCs in model systems during heating [31]. This implies that the effect of sugar varies according to the anthocyanidins that make up the extract.

### 3.3. Sensory Evaluation Scores of RS

**Figure 4:**
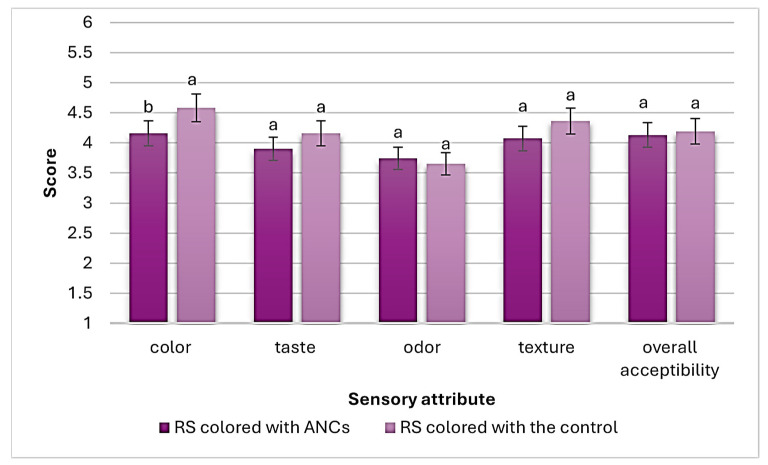
Sensory evaluation scores of RS (colored with ANCs or the control). Values are presented as mean ± SD (*n* = 3). Significant differences (*p* < 0.05) are indicated in different letters (a, b).

Figure 4 shows the sensory evaluation scores (out of 5) of RS (colored with ANCs and the control). As a result, RS colored with ANCs received a significantly (*p* < 0.05) lower color score than that colored with the control. However, no significant (*p* ≥ 0.05) differences in taste, texture, odor, and overall acceptability scores were found between RS colored with ANCs and the control.

It is well known that synthetic colorants have higher tinctorial strength than natural colorants; the replacement of synthetic dyes with natural pigments may also be limited by the inability of natural pigments to match the color characteristics of synthetic colorants [32], which might be a reason why the color of the control was favored over ANCs in the present study.

Furthermore, although many natural colorants can impart undesirable flavors and aromas to products, such as those from red radishes or red beets [32], RS colored with ANCs did not suffer from any undesirable taste or odor when compared to the control.

Similarly, Batu and Arslan [13] prepared RS with either sour cherry or black grape juice concentrates and evaluated its sensory properties in terms of appearance, color, odor, and overall acceptability. Their results showed that grape and cherry received color scores of 3.65–3.91 out of 5 and 3.51–3.91 out of 5, respectively, which were lower than the color score of RS colored with either ANCs or the control in the present study. On the other hand, their odor score reached 4 out of 5 points, which was higher than that obtained for RS prepared with grape ANCs or the control.

Unlike the results of the present study, the sensory evaluation of hard candy and sweet jelly colored with either ANCs from purple carrots or Carmine (control) showed no significant (*p* < 0.05) differences in color scores between hard candy and sweet jelly with 0.30% ANCs and the control with 0.1% Carmine. Hard candy and sweet jelly prepared with 0.5% ANCs recorded lower color scores than those with Carmine, similar to the current work’s findings. There were significant (*p* < 0.05) differences in taste and odor scores between hard candy and sweet jelly prepared by adding different levels of ANCs from purple carrots and the control with 0.10% Carmine [33], which contradicts the results of the present study.

## 4. Conclusions

In this study, the spectral properties of purified ANCs extracted from red grape pomace were investigated in acidic, neutral, and alkaline buffer systems. Grape ANCs expressed an intense red color in acidic environments, where multiple bathochromic shifts in λ_max_ caused the color to shift from red to violet to blue as the pH increased. On the other hand, RS was successfully colored with ANCs, although the half-life of the unpurified ANCs revealed that they were more stable than the purified ones. Therefore, instead of being used in RS, purified ANCs could be useful as a colorant in other food systems. Furthermore, taste, odor, and other sensory qualities were unaffected, even though the control’s color was superior to that of the ANCs. Hence, grape ANCs could provide the food industry with a natural pigment as a viable substitute for synthetic dyes, particularly in food products with a high sugar content and limited shelf life.

## Figures and Tables

**Table 1:** Formulation of RS preparation.

Ingredients	(wt/wt ^1^) %
Water	46.3
Sugar	37.04
Starch	9.26
Citric acid	0.46
Rose water	2.31
Colorant (control or ANCs)	4.63

^1^ wt = weight.

**Table 2:** Spectral properties of ANCs at different pH values.

pH	λ_max_ of ANCs ^1^	Relative Absorbance% of ANCs
2	517	100
3	517	71.9
4	519	41.7
5	521	34.5
6	535	46.9
7	557	44.9
8	581	46.7
9	590	56.1

^1^ ANCs: anthocyanins.

**Table 3:** Degradation kinetics parameters of ANCs and the control in RS during storage.

Sample	K	t ½
RS with unpurified ANCs ^1^	7.2 × 10^−3^	96.25
RS with purified ANCs	2.42 × 10^−2^	28.64
RS with the control	NA	NA

^1^ ANCs: anthocyanins, NA: not applicable.

## Data Availability

The original contributions presented in this study are included in the article; further inquiries can be directed to the corresponding authors.
